# Supplementary material for: Pathologic polyglutamine aggregation begins with a self-poisoning polymer crystal
Source: bioRxiv. 2023 Jul 29:2023.03.20.533418. Originally published 2023 Mar 21. Preprint. [Version 2] doi: 10.1101/2023.03.20.533418 (PMC10055281; doi:10.1101/2023.03.20.533418)
Supplement: Supplement 1 — Supplemental Files Table S1. List of plasmids and sequences. Table S2. Amyloid predictor output. Figure S1. A. DAmFRET plots of polyQ length variants. Labels above the boxed regions of Q35 and Q40 in [PIN+] indicate the percentage of cells in the high-FRET region, revealing infrequent but significant nucleation for the latter (p = 0.004, one-tailed T-test). Shown are representative plots of biological triplicates. B. DAmFRET plots of polypeptides composed of tandem repeats of q (subscripted) Qs separated by an N for a total length of 60 residues. Plots are representative of biological triplicates. C. DAmFRET plots of polypeptides composed of tandem repeats of the indicated N-rich sequences, for a total length of 60 residues, showing negligible nucleation in the absence of a conformational template. Note that because the nominal pattern repeats, “Q1N2”, “Q1N3”, and “Q1N4” are synonymous to “N2Q1”, “N3Q1”, and “N4Q1”, respectively. Plots are representative of biological triplicates. D. DAmFRET plots of polypeptides composed of tandem repeats of the indicated sequences, for a total length of 60 residues, showing that Q3X and Q5X have a greater amyloid propensity than Q4X regardless of the identity of X. Labels above the boxed regions of the [PIN+] Q4N, Q4G, and Q4H plots indicate the percentage of cells in the high-FRET region, revealing rare but significant nucleation for the latter (p = 0.046 versus Q4N, one-tailed T-test). Plots are representative of biological triplicates. Figure S2. A. Molecular simulations of model Q zippers formed by a pair of two-stranded antiparallel β-sheets, containing a single serine residue (QQQSQQQ) per strand. The structure is unstable when the S side chains face inward (top), but not when the S side chains face outward (bottom). B. Simulations of model steric zippers formed by a pair of four-stranded antiparallel β-sheets, containing a single asparagine (top) or serine (bottom) residue per strand. The structure proved less stable in the [file NIHPP2023.03.20.533418v2-supplement-1.pdf]

bioRxiv preprint doi: https://doi.org/10.1101/2023.03.20.533418; this version posted July 29, 2023. The copyright holder for this preprint (which was not certified by peer review) is the author/funder, who has granted bioRxiv a license to display the preprint in perpetuity. It is made available under aCC-BY 4.0 International license.

Supplemental Files

Table S1. List of plasmids and sequences.

Table S2. Amyloid predictor output.

bioRxiv preprint doi: https://doi.org/10.1101/2023.03.20.533418; this version posted July 29, 2023. The copyright holder for this preprint (which was not certified by peer review) is the author/funder, who has granted bioRxiv a license to display the preprint in perpetuity. It is made available under aCC-BY 4.0 International license.

Figure S1

A

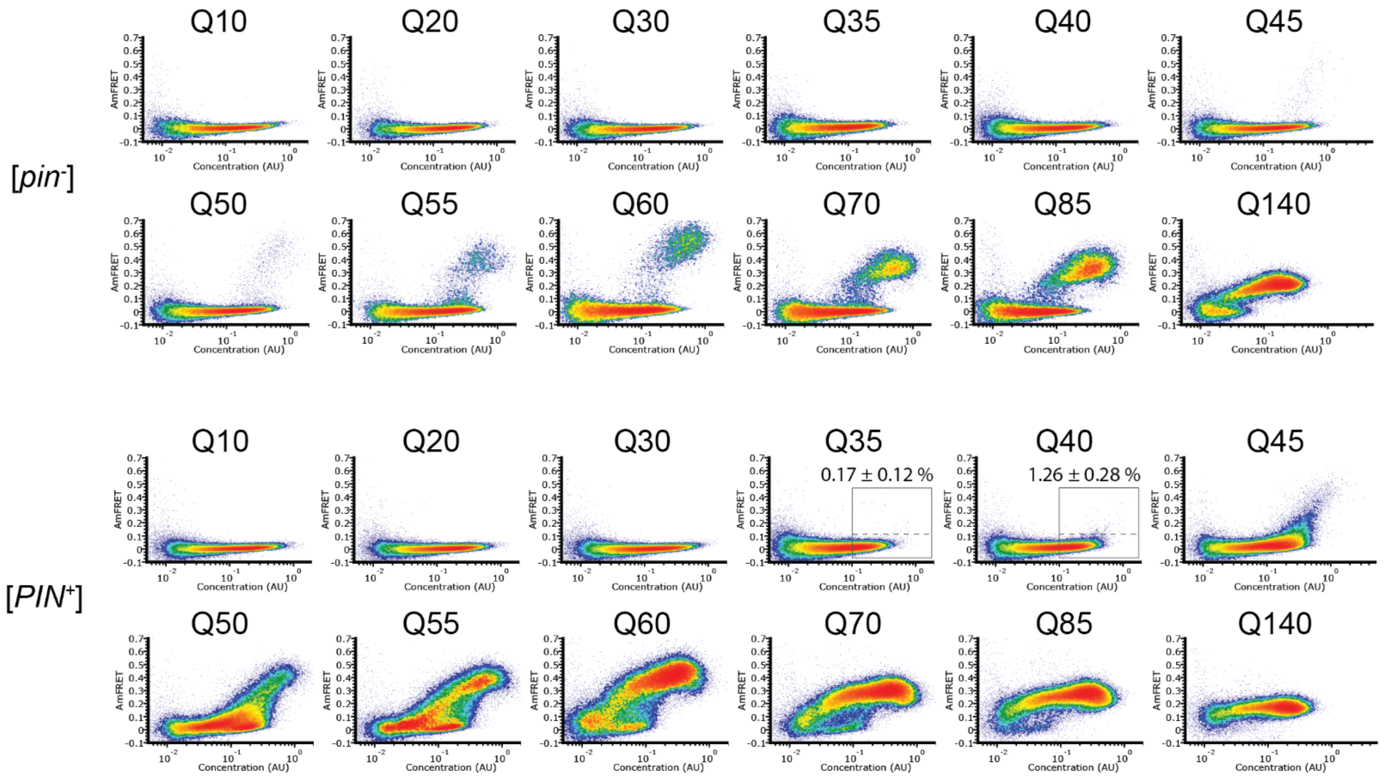

B

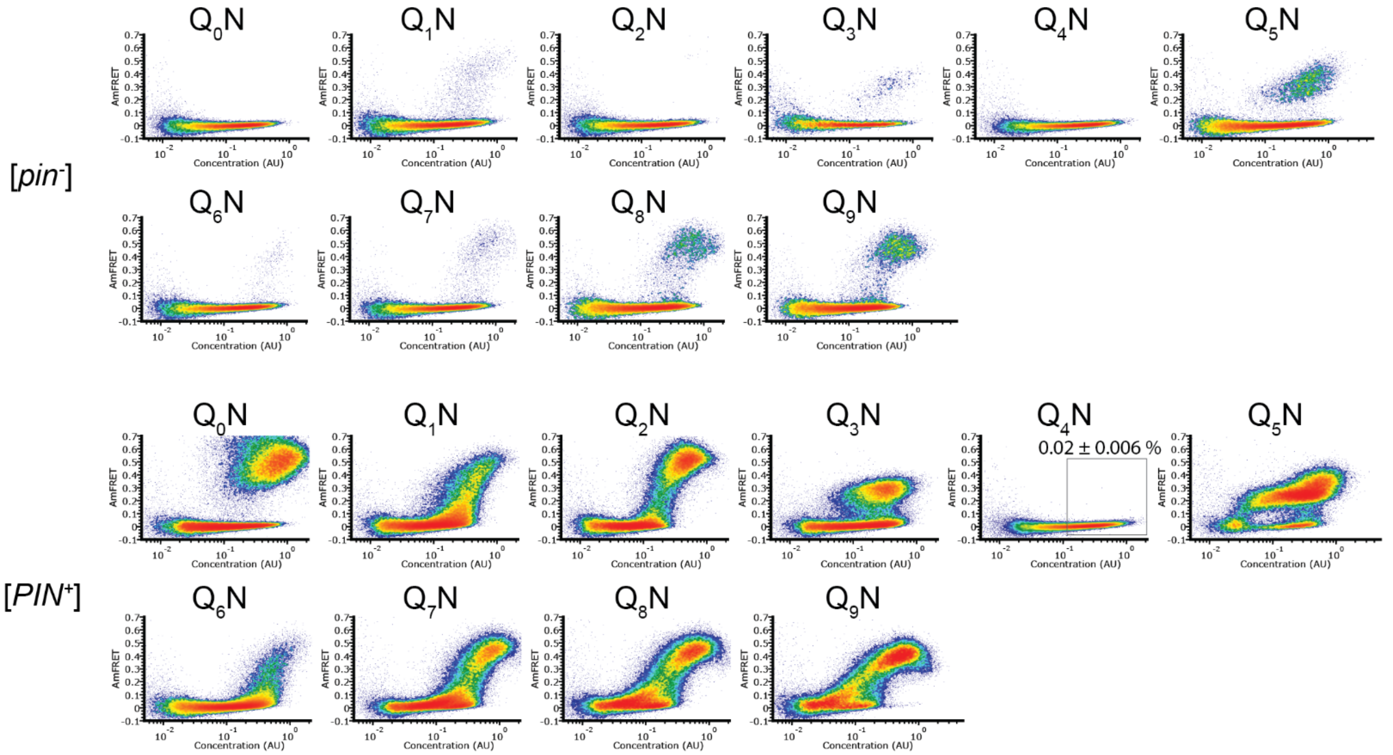

bioRxiv preprint doi: https://doi.org/10.1101/2023.03.20.533418; this version posted July 29, 2023. The copyright holder for this preprint (which was not certified by peer review) is the author/funder, who has granted bioRxiv a license to display the preprint in perpetuity. It is made available under aCC-BY 4.0 International license.

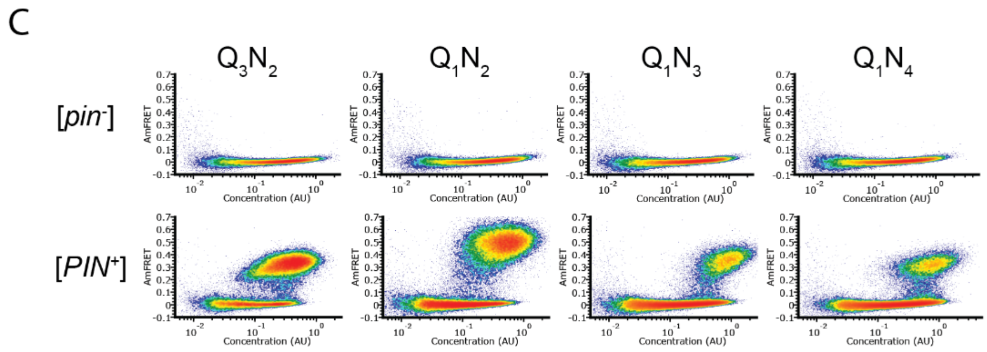

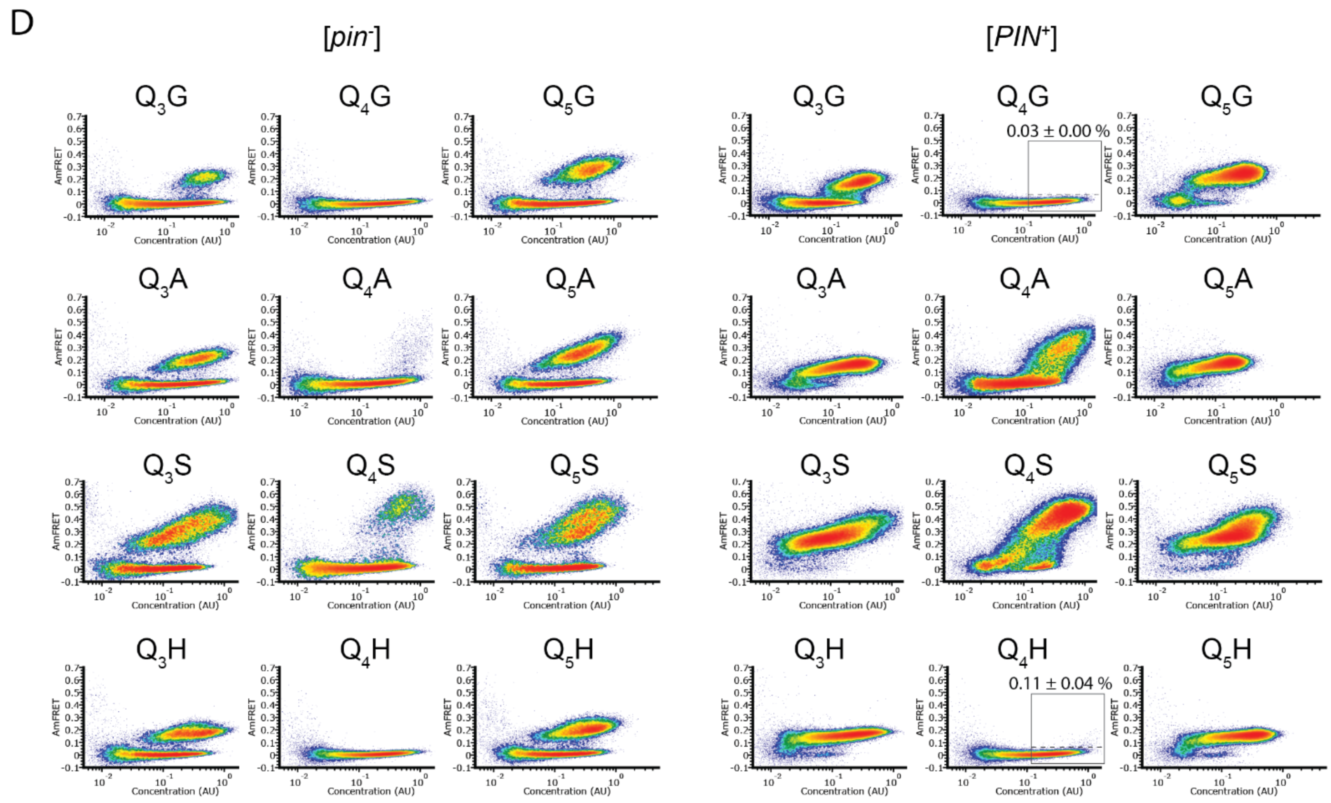

bioRxiv preprint doi: https://doi.org/10.1101/2023.03.20.533418; this version posted July 29, 2023. The copyright holder for this preprint (which was not certified by peer review) is the author/funder, who has granted bioRxiv a license to display the preprint in perpetuity. It is made available under aCC-BY 4.0 International license.

Figure S1.

A. DAmFRET plots of polyQ length variants. Labels above the boxed regions of Q35 and Q40 in [*PIN*$^+$] indicate the percentage of cells in the high-FRET region, revealing infrequent but significant nucleation for the latter ($p = 0.004$, one-tailed T-test). Shown are representative plots of biological triplicates.
B. DAmFRET plots of polypeptides composed of tandem repeats of *q* (subscripted) Qs separated by an N for a total length of 60 residues. Plots are representative of biological triplicates.
C. DAmFRET plots of polypeptides composed of tandem repeats of the indicated N-rich sequences, for a total length of 60 residues, showing negligible nucleation in the absence of a conformational template. Note that because the nominal pattern repeats, "Q_1N_2", "Q_1N_3", and "Q_1N_4" are synonymous to "N_2Q_1", "N_3Q_1", and "N_4Q_1", respectively. Plots are representative of biological triplicates.
D. DAmFRET plots of polypeptides composed of tandem repeats of the indicated sequences, for a total length of 60 residues, showing that Q_3X and Q_5X have a greater amyloid propensity than Q_4X regardless of the identity of X. Labels above the boxed regions of the [*PIN*$^+$] Q_4N, Q_4G, and Q_4H plots indicate the percentage of cells in the high-FRET region, revealing rare but significant nucleation for the latter ($p = 0.046$ versus Q_4N, one-tailed T-test). Plots are representative of biological triplicates.

bioRxiv preprint doi: https://doi.org/10.1101/2023.03.20.533418; this version posted July 29, 2023. The copyright holder for this preprint (which was not certified by peer review) is the author/funder, who has granted bioRxiv a license to display the preprint in perpetuity. It is made available under aCC-BY 4.0 International license.

Figure S2

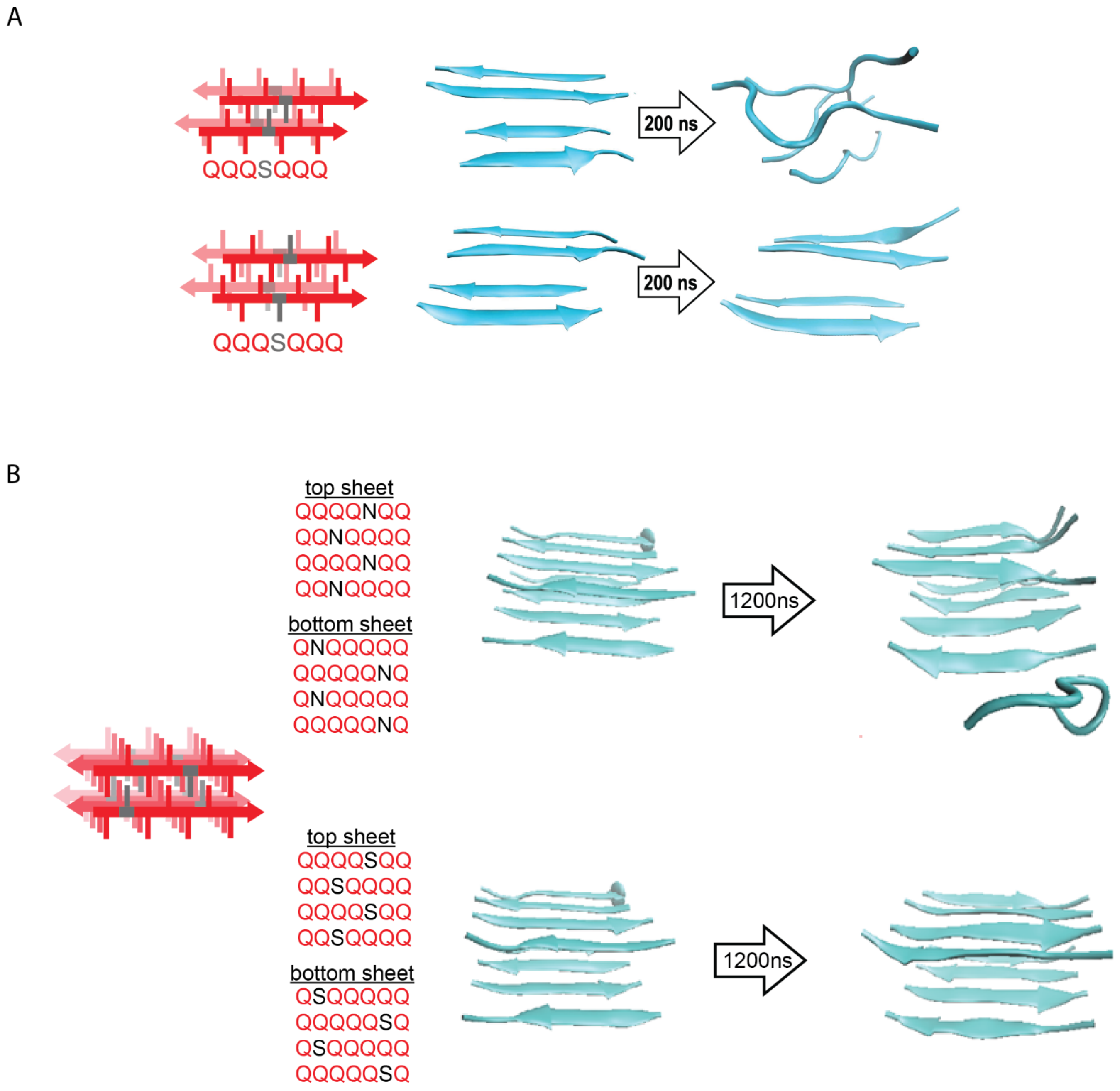

bioRxiv preprint doi: https://doi.org/10.1101/2023.03.20.533418; this version posted July 29, 2023. The copyright holder for this preprint (which was not certified by peer review) is the author/funder, who has granted bioRxiv a license to display the preprint in perpetuity. It is made available under aCC-BY 4.0 International license.

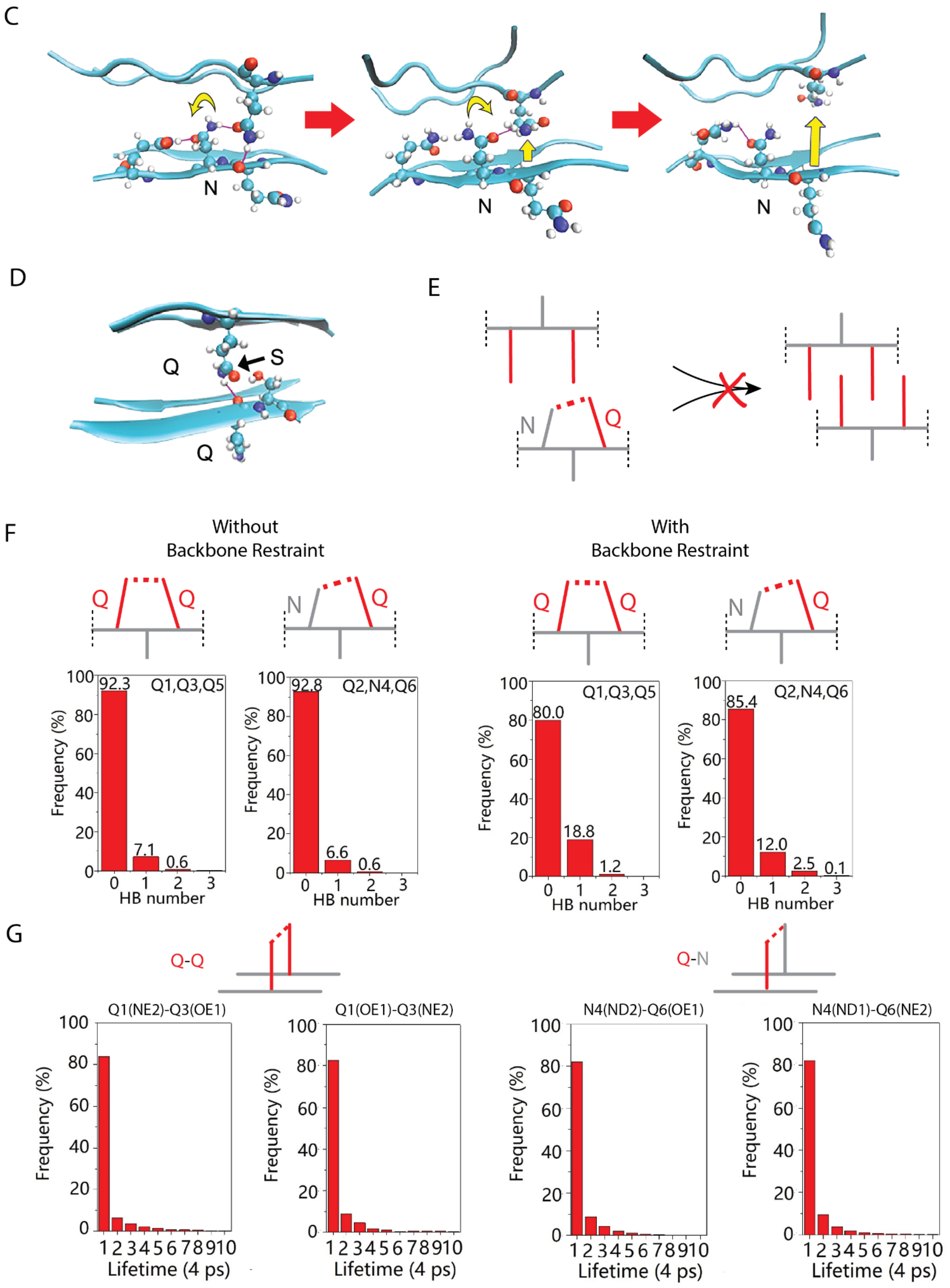

bioRxiv preprint doi: https://doi.org/10.1101/2023.03.20.533418; this version posted July 29, 2023. The copyright holder for this preprint (which was not certified by peer review) is the author/funder, who has granted bioRxiv a license to display the preprint in perpetuity. It is made available under aCC-BY 4.0 International license.

Figure S2.

A. Molecular simulations of model Q zippers formed by a pair of two-stranded antiparallel β-sheets, containing a single serine residue (QQQSQQQ) per strand. The structure is unstable when the S side chains face inward (top), but not when the S side chains face outward (bottom).
B. Simulations of model steric zippers formed by a pair of four-stranded antiparallel β-sheets, containing a single asparagine (top) or serine (bottom) residue per strand. The structure proved less stable in the case of asparagine.
C. As a consequence of the N side chain's interception of the opposing Q side chain's H-bond, the Q is no longer anchored in the outstretched configuration and sterically interferes with the ordering of adjacent Qs. This effect propagates through the zipper, resulting in its dissolution.
D. As for N, the side chain of S is too short to H-bond with the opposing backbone. Unlike for N, however, the S side chain is also too short to intercept the opposing Q side chain's H-bond, allowing the Q to H-bond (black arrow) the backbone amide adjacent to the S. Therefore, whereas Q zippers cannot accommodate internal N residues, they can accommodate sparse internal S residues.
E. Schematic demonstrating how a polar clasp (red dashed line) would preclude Q zipper formation.
F. Schema and frequencies of polar clasps occurring between two unilaterally adjacent Q side chains (left) or a unilaterally adjacent N and Q side chain (right) within a QQQNQQQ peptide, simulated either with (top) or without (bottom) the backbone restrained in a β conformation. The bar graphs show that polar clasps between Q and N occur less frequently than between Q and Q, indicating that the mechanism of Q zipper destabilization by N side chains cannot be attributed to polar clasps.
G. Schema and lifetimes of H-bonds between exterior stacked (axially adjacent) Q side chains (top) or N and Q side chains (bottom) in the Q zipper simulated in **Fig. 2D**, showing no difference in stabilities between axial H-bonds between Q and Q/N side chains.

bioRxiv preprint doi: https://doi.org/10.1101/2023.03.20.533418; this version posted July 29, 2023. The copyright holder for this preprint (which was not certified by peer review) is the author/funder, who has granted bioRxiv a license to display the preprint in perpetuity. It is made available under aCC-BY 4.0 International license.

Figure S3

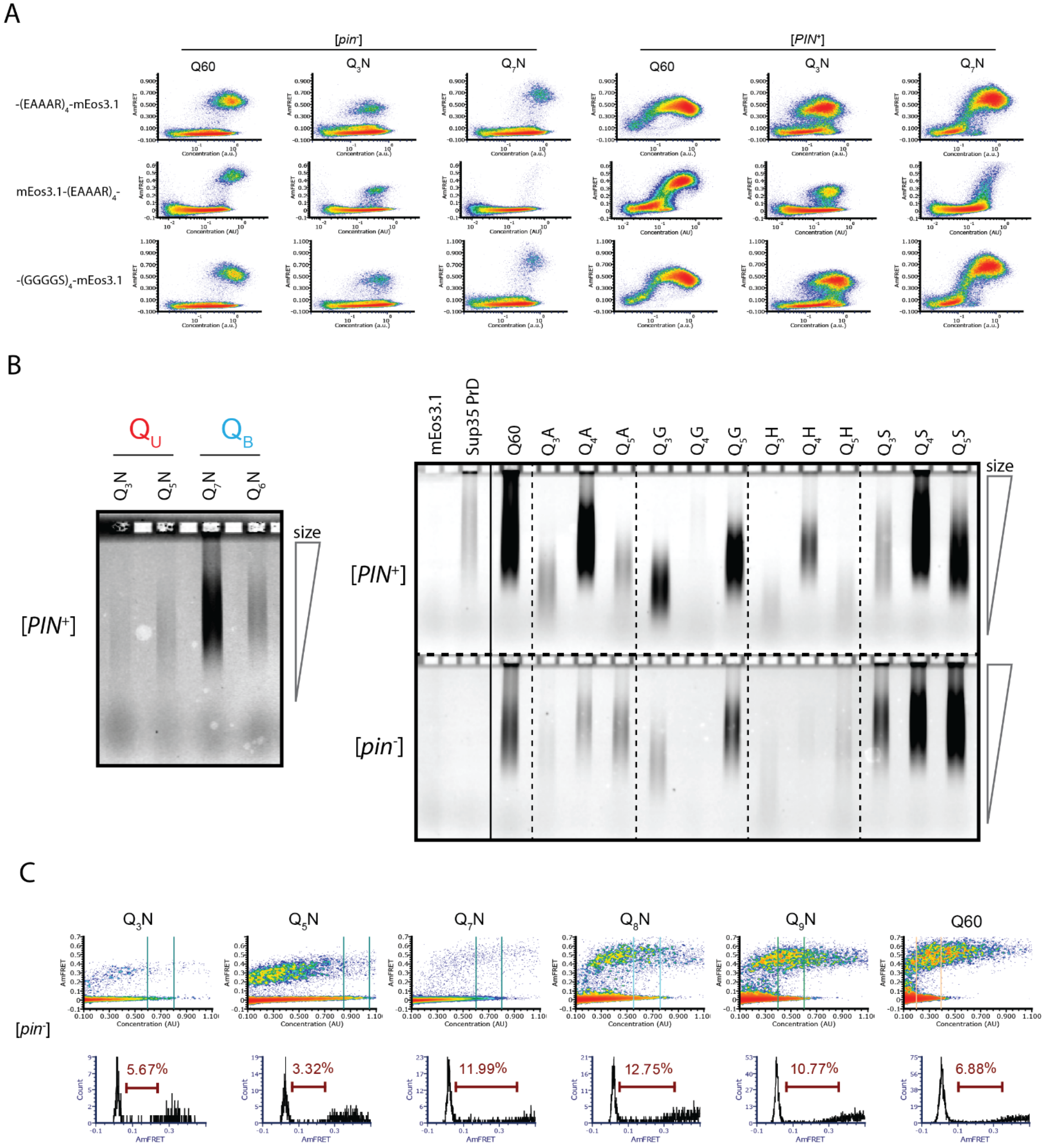

bioRxiv preprint doi: https://doi.org/10.1101/2023.03.20.533418; this version posted July 29, 2023. The copyright holder for this preprint (which was not certified by peer review) is the author/funder, who has granted bioRxiv a license to display the preprint in perpetuity. It is made available under aCC-BY 4.0 International license.

Figure S3.

A. DAmFRET plots of the indicated sequence variants with either a C-terminal $(EAAAR)_4$-mEos3.1 fusion, as used throughout this work, an N-terminal mEos3.1-$(EAAAR)_4$ fusion, or a C-terminal $(GGGGS)_4$-mEos3.1 fusion, showing that the sequence-specific differences in relative steady state AmFRET levels do not depend on the linker or terminus fused. Plots are representative of biological triplicates.
B. Fluorescence images of SDD-AGE gels showing the size distributions of SDS-resistant complexes of the indicated mEos3.1-tagged proteins. Left: raw data quantified in **Fig. 3C**. Right: Additional Q_3X, Q_4X, Q_5X proteins, showing that Q_U amyloids are consistently smaller than other amyloids, such as those of Q60 and Sup35 PrD (which only nucleates in [*PIN*$^+$] cells). The solid line shows where the image was spliced, although all lanes are from the same gel. Lysates were normalized by fluorescence to within 50% of each other prior to loading.
C. Histograms of AmFRET values for the indicated gates (at the respective approximate EC50s) for the indicated sequences. The brown gate on the histograms shows the percentage of transitioning cells.

bioRxiv preprint doi: https://doi.org/10.1101/2023.03.20.533418; this version posted July 29, 2023. The copyright holder for this preprint (which was not certified by peer review) is the author/funder, who has granted bioRxiv a license to display the preprint in perpetuity. It is made available under aCC-BY 4.0 International license.

Figure S4

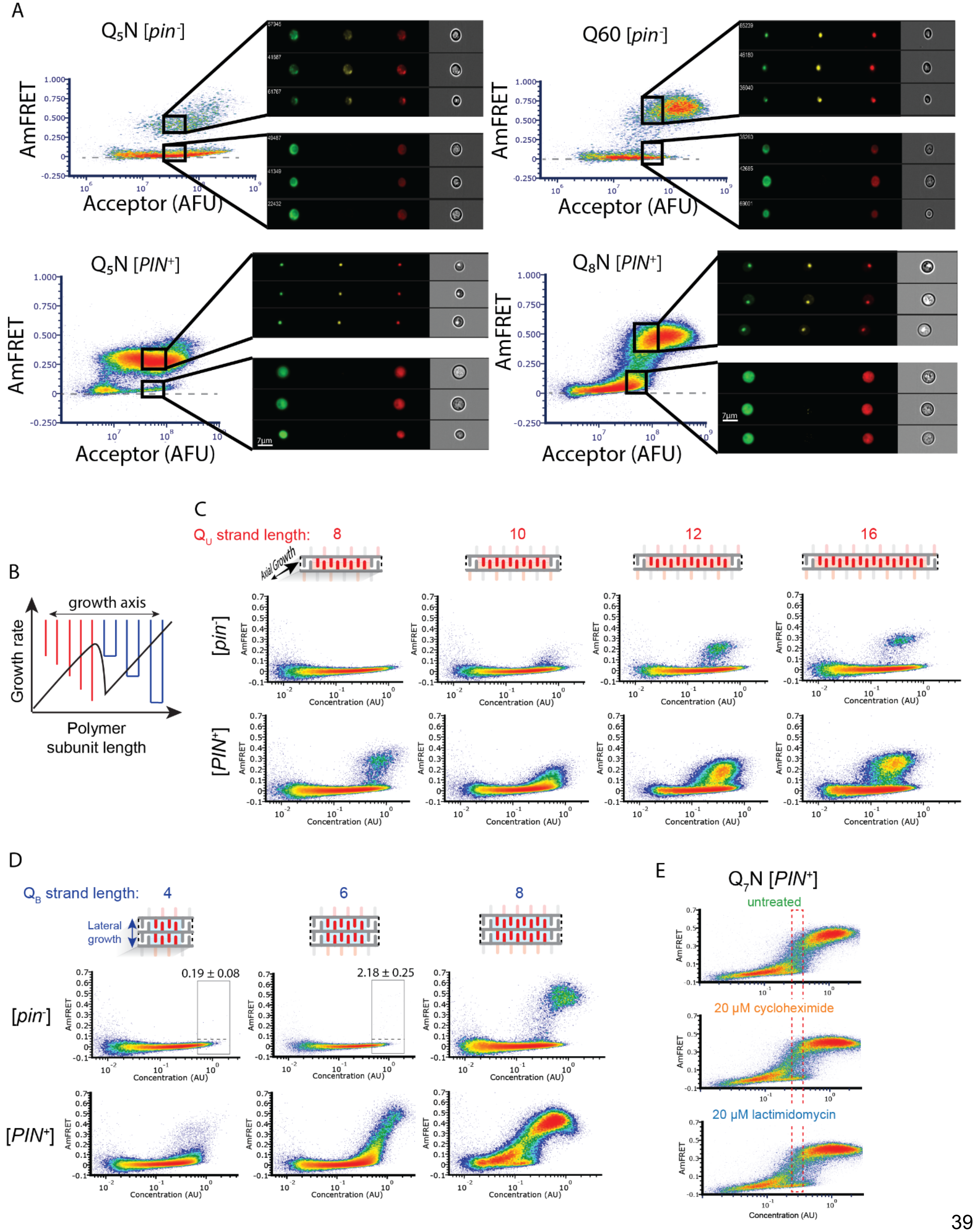

bioRxiv preprint doi: https://doi.org/10.1101/2023.03.20.533418; this version posted July 29, 2023. The copyright holder for this preprint (which was not certified by peer review) is the author/funder, who has granted bioRxiv a license to display the preprint in perpetuity. It is made available under aCC-BY 4.0 International license.

Figure S4.

A. DAmFRET plots of Q_5N, Q60, and Q_8N acquired using imaging flow cytometry, showing gates at high expression for both high- or low-AmFRET populations. Insets show from left to right the distribution of donor, FRET, and acceptor fluorescence, respectively, in representative cells from each gate.
B. Schematic of the rate of polymer crystallization as a function of length, showing a sharp deceleration when the polymer length is equally compatible with either of two polymorphs. Adapted from (Ungar et al., 2005).
C. DAmFRET plots of [*pin*$^-$] cells expressing unilateral contiguity variants of the Q_3N base sequence, showing that at least five unilaterally contiguous glutamines (see schematic) are required for *de novo* nucleation of single long Q zipper amyloids. Plots are representative of biological triplicates.
D. DAmFRET plots of bilateral contiguity variants (Q_4N_2, Q_6N_2, Q_8N_2), showing that at least six bilaterally contiguous Qs are required for *de novo* amyloid formation. Numbers indicate the percentage of cells in the high-FRET boxed region, revealing significant nucleation by Q_6N_2 ($p = 0.0004$, T-test).
E. DAmFRET plots of Q_7N in [*pin*$^-$] cells treated as indicated for six hours prior to analysis. The boxed region was used to compute histograms of AmFRET in Fig. 4E.

bioRxiv preprint doi: https://doi.org/10.1101/2023.03.20.533418; this version posted July 29, 2023. The copyright holder for this preprint (which was not certified by peer review) is the author/funder, who has granted bioRxiv a license to display the preprint in perpetuity. It is made available under aCC-BY 4.0 International license.

Figure S5

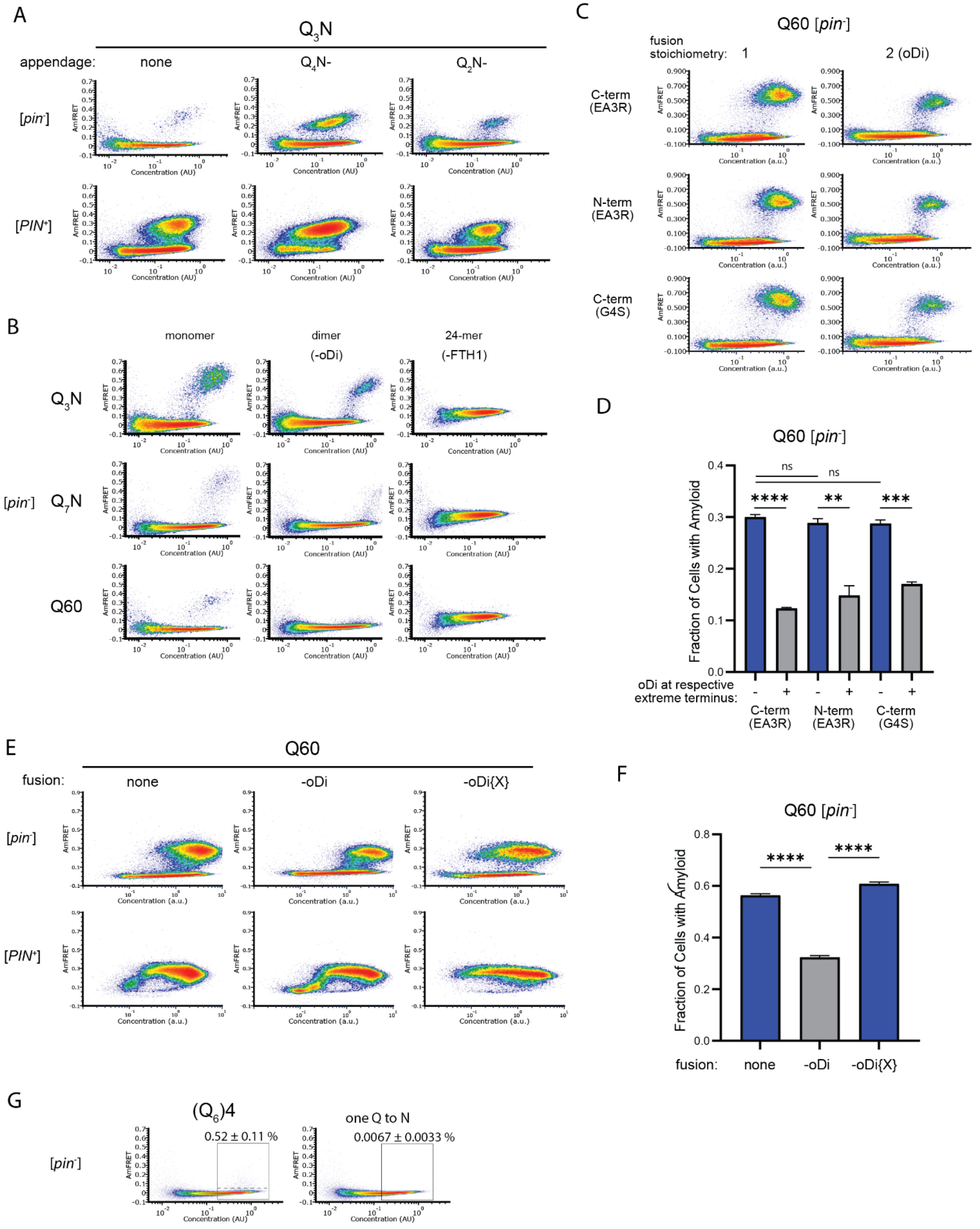

bioRxiv preprint doi: https://doi.org/10.1101/2023.03.20.533418; this version posted July 29, 2023. The copyright holder for this preprint (which was not certified by peer review) is the author/funder, who has granted bioRxiv a license to display the preprint in perpetuity. It is made available under aCC-BY 4.0 International license.

Figure S5.

A. DAmFRET plots of cells expressing Q_3N (length 60) with the indicated appendage (length 30). Plots are representative of biological triplicates.
B. DAmFRET plots of [*pin*$^-$] and [*PIN*$^+$] cells expressing the indicated sequences either unfused ("monomer") or fused to oDi ("dimer") for FTH1 (24-mer). Plots are representative of biological triplicates.
C. DAmFRET plots of cells expressing Q60 either with or without oDi and with the indicated linkers and termini of the fusion. Plots are representative of biological triplicates.
D. Quantification of the data in C), showing that oDi reduces Q60 nucleation irrespective of the linker and terminus it is fused to. Shown are means +/- SEM. **, ***, **** $p < 0.01$, < 0.001, < 0.0001; t-test.
E. DAmFRET plots of cells expressing Q60 either with or without oDi or a monomeric mutant of oDi (oDi{X}). Plots are representative of biological triplicates.
F. Quantification of the data in E), showing that the monomerizing mutation eliminates the amyloid-inhibiting effect of oDi. Shown are means +/- SEM. **** $p < 0.0001$; t-test.
G. DAmFRET plots of [*pin*$^-$] cells expressing a synthetic minimal polyQ amyloid-forming sequence, or the same sequence with the tenth Q mutated to N. Quantitation is the same as in Fig. 5C. Plots are representative of biological triplicates.

Movie S1. Simulation of the pure Q zipper shown in **Fig. 2D** (top model), showing its persistence.

Movie S2. Simulation of the N-substituted Q zipper shown in **Fig. 2D** (bottom model), showing the motions of side chains summarized in **Fig. S2C**.

Figure panel	plasmid name	Annotated Sequence	extra N-term tag	linker	N-term Tag	N-term linker	Inserted Peptide Sequence	linker	C-term Tag	linker	extra tag
3 C; S3 B	rhx0935	mEos3.1							mEos3.1		
3 C; S3 B	rhx0974	Sup35 PrD					MSDSNQGNNQ QNYQQYSQNG NQQQGNNRYQ GYQAYNAQAQ PAGGYYQNYQ GYSGYQQGGY QQYNPDAGYQ QQYNPQGGYQ QYNPQGGYQQ QFNPQGGRGN YKNFNYNNNL QGYQAGFQPQ SQGMSLNDFQ KQQKQ	EAAAREAAAR EAAAREAAAR	mEos3.1		
1 D	rhx1177a	Q85					QQQQQQQQQQ QQQQQQQQQQ QQQQQQQQQQ QQQQQQQQQQ QQQQQQQQQQ QQQQQQQQQQ QQQQQQQQQQ QQQQQQQQQQ QQQ	EAAAREAAAR EAAAREAAAR	mEos3.1		
1 D	rhx1177d	Q20					QQQQQQQQQQ QQQQQQQQQQ	EAAAREAAAR EAAAREAAAR	mEos3.1		
1 D	rhx1177e	Q30					QQQQQQQQQQ QQQQQQQQQQ QQQQQQQQQQ	EAAAREAAAR EAAAREAAAR	mEos3.1		
1 D	rhx1177f	Q70					QQQQQQQQQQ QQQQQQQQQQ QQQQQQQQQQ QQQQQQQQQQ QQQQQQQQQQ QQQQQQQQQQ QQQQQQQQ	EAAAREAAAR EAAAREAAAR	mEos3.1		
1 D	rhx1177g	Q10					QQQQQQQQQQ	EAAAREAAAR EAAAREAAAR	mEos3.1		
1 D	rhx1177h	Q55					QQQQQQQQQQ QQQQQQQQQQ QQQQQQQQQQ QQQQQQQQQQ QQQQQQQQQQ QQQQ	EAAAREAAAR EAAAREAAAR	mEos3.1		
1 D	rhx1177j	Q140					QQQQQQQQQQ QQQQQQQQQQ QQQQQQQQQQ QQQQQQQQQQ QQQQQQQQQQ QQQQQQQQQQ QQQQQQQQQQ QQQQQQQQQQ QQQQQQQQQQ QQQQQQQQQQ QQQQQQQQQQ QQQQQQQQQQ QQQQQQQQQQ QQQQQQQQQQ	EAAAREAAAR EAAAREAAAR	mEos3.1		
1 D	rhx1177l	Q35					QQQQQQQQQQ QQQQQQQQQQ QQQQQQQQQQ QQQQ	EAAAREAAAR EAAAREAAAR	mEos3.1		
1 D	rhx1177m	Q40					QQQQQQQQQQ QQQQQQQQQQ QQQQQQQQQQ QQQQQQQQQ	EAAAREAAAR EAAAREAAAR	mEos3.1		
1 D	rhx1177n	Q45					QQQQQQQQQQ QQQQQQQQQQ QQQQQQQQQQ QQQQQQQQQQ QQQQ	EAAAREAAAR EAAAREAAAR	mEos3.1		
1 D	rhx1177o	Q50					QQQQQQQQQQ QQQQQQQQQQ QQQQQQQQQQ QQQQQQQQQQ QQQQQQQQQ	EAAAREAAAR EAAAREAAAR	mEos3.1		
1 E; 3 B,D; 4 A,D	rhx2602	Q_3N					QQQNQQQNQQ QNQQQNQQQN QQQNQQQNQQ QNQQQNQQQN QQQNQQQNQQ QNQQQNQQQN	EAAAREAAAR EAAAREAAAR	mEos3.1		
1 E	rhx2603	Q_4N					QQQQNQQQQN QQQQNQQQQN QQQQNQQQQN QQQQNQQQQN QQQQNQQQQN QQQQNQQQQ	EAAAREAAAR EAAAREAAAR	mEos3.1		
1 E; 3 B,D; S4 A	rhx2604	Q_5N					QQQQQNQQQQ QNQQQQQNQQ QQQNQQQQQN QQQQQNQQQQ QNQQQQQNQQ QQQNQQQQQN	EAAAREAAAR EAAAREAAAR	mEos3.1		
3 B,D; 4 A,D,E	rhx2605	Q_7N					QQQQQQQNQQ QQQQQNQQQQ QQQNQQQQQQ QNQQQQQQQN QQQQQQQNQQ QQQQQNQQQQ	EAAAREAAAR EAAAREAAAR	mEos3.1		
4 A,D	rhx2606	Q_9N					QQQQQQQQQN QQQQQQQQQN QQQQQQQQQN QQQQQQQQQN QQQQQQQQQN QQQQQQQQQ	EAAAREAAAR EAAAREAAAR	mEos3.1		
1 C,E	rhx2681	N60/ Q_0N					NNNNNNNNNN NNNNNNNNNN NNNNNNNNNN NNNNNNNNNN NNNNNNNNNN NNNNNNNNN	EAAAREAAAR EAAAREAAAR	mEos3.1		
1 C; 4 A,B,D	rhx2682	Q60					QQQQQQQQQQ QQQQQQQQQQ QQQQQQQQQQ QQQQQQQQQQ QQQQQQQQQQ QQQQQQQQQ	EAAAREAAAR EAAAREAAAR	mEos3.1		
1 E	rhx2683	Q_1N					QNQNQNQNQN QNQNQNQNQN QNQNQNQNQN QNQNQNQNQN QNQNQNQNQN QNQNQNQNQ	EAAAREAAAR EAAAREAAAR	mEos3.1		
1 E	rhx2684	Q_2N					QQNQQNQQNQ QNQQNQQNQQ NQQNQQNQQN QQNQQNQQNQ QNQQNQQNQQ NQQNQQNQQ	EAAAREAAAR EAAAREAAAR	mEos3.1		
S3 B; 4 A,D	rhx2685	Q_6N					QQQQQQNQQQ QQQNQQQQQQ NQQQQQQNQQ QQQQNQQQQQ QNQQQQQQNQ QQQQQNQQQ	EAAAREAAAR EAAAREAAAR	mEos3.1		
S1 C	rhx2686	Q_1N_3					NNNQNNNQNN NQNNNQNNNQ NNNQNNNQNN NQNNNQNNNQ NNNQNNNQNN NQNNNQNNN	EAAAREAAAR EAAAREAAAR	mEos3.1		
S1 C	rhx2687	Q_1N_4					NNNNQNNNNQ NNNNQNNNNQ NNNNQNNNNQ NNNNQNNNNQ NNNNQNNNNQ NNNNQNNNN	EAAAREAAAR EAAAREAAAR	mEos3.1		
3 B,D; S3 B,C; 4 A,D	rhx3071	Q_8N					QQQQQQQQNQ QQQQQQQNQQ QQQQQQNQQQ QQQQQNQQQQ QQQQNQQQQQ QQQNQQQQQQ	EAAAREAAAR EAAAREAAAR	mEos3.1		
1 E	rhx3117	Q_3S					QQQSQQQSQQ QSQQQSQQQS QQQSQQQSQQ QSQQQSQQQS QQQSQQQSQQ QSQQQSQQQ	EAAAREAAAR EAAAREAAAR	mEos3.1		
1 E	rhx3118	Q_4S					QQQQSQQQQS QQQQSQQQQS QQQQSQQQQS QQQQSQQQQS QQQQSQQQQS QQQQSQQQQ	EAAAREAAAR EAAAREAAAR	mEos3.1		
1 E	rhx3119	Q_5S					QQQQQSQQQQ QSQQQQQSQQ QQQSQQQQQS QQQQQSQQQQ QSQQQQQSQQ QQQSQQQQQ	EAAAREAAAR EAAAREAAAR	mEos3.1		
1 E; 3 C	rhx3265	Q_3A					QQQAQQQAQQ QAQQQAQQQA QQQAQQQAQQ QAQQQAQQQA QQQAQQQAQQ QAQQQAQQQ	EAAAREAAAR EAAAREAAAR	mEos3.1		
1 E; 3 C	rhx3266	Q_4A					QQQQAQQQQA QQQQAQQQQA QQQQAQQQQA QQQQAQQQQA QQQQAQQQQA QQQQAQQQQ	EAAAREAAAR EAAAREAAAR	mEos3.1		
1 E; 3 C	rhx3267	Q_5A					QQQQQAQQQQ QAQQQQQAQQ QQQAQQQQQA QQQQQAQQQQ QAQQQQQAQQ QQQAQQQQQ	EAAAREAAAR EAAAREAAAR	mEos3.1		
1 E	rhx4276	Q_3H					QQQHQQQHQQ QHQQQHQQQH QQQHQQQHQQ QHQQQHQQQH QQQHQQQHQQ QHQQQHQQQH QQQHQQQHQQ QHQ	EAAAREAAAR EAAAREAAAR	mEos3.1		
1 E	rhx4277	Q_4H					QQQQHQQQQH QQQQHQQQQH QQQQHQQQQH QQQQHQQQQH QQQQHQQQQH QQQQHQQQQH QQQQHQQQQH QQQ	EAAAREAAAR EAAAREAAAR	mEos3.1		
1 E	rhx4278	Q_5H					QQQQQHQQQQ QHQQQQQHQQ QQQHQQQQQH QQQQQHQQQQ QHQQQQQHQQ QQQHQQQQQH QQQQQHQQQQ QHQ	EAAAREAAAR EAAAREAAAR	mEos3.1		
1 E	rhx3453	Q_3G					QQQGQQQGQQ QGQQQGQQQG QQQGQQQGQQ QGQQQGQQQG QQQGQQQGQQ QGQQQGQQQ	EAAAREAAAR EAAAREAAAR	mEos3.1		
1 E	rhx3454	Q_4G					QQQQGQQQQG QQQQGQQQQG QQQQGQQQQG QQQQGQQQQG QQQQGQQQQG QQQQGQQQQ	EAAAREAAAR EAAAREAAAR	mEos3.1		
1 E	rhx3455	Q_5G					QQQQQGQQQQ QGQQQQQGQQ QQQGQQQQQG QQQQQGQQQQ QGQQQQQGQQ QQQGQQQQQ	EAAAREAAAR EAAAREAAAR	mEos3.1		
S3 A	rhx3989	Q60			mEos3.1	EAAAREAAAR EAAAREAAAR	QQQQQQQQQQ QQQQQQQQQQ QQQQQQQQQQ QQQQQQQQQQ QQQQQQQQQQ QQQQQQQQQ				
S3 A	rhx4710	Q_3N			mEos3.1	EAAAREAAAR EAAAREAAAR	QQQNQQQNQQ QNQQQNQQQN QQQNQQQNQQ QNQQQNQQQN QQQNQQQNQQ QNQQQNQQQ				
S3 A	rhx4068	Q_7N			mEos3.1	EAAAREAAAR EAAAREAAAR	QQQQQQQNQQ QQQQQNQQQQ QQQNQQQQQQ QNQQQQQQQN QQQQQQQNQQ QQQQQNQQQ				
S3 A	rhx4584	Q60					QQQQQQQQQQ QQQQQQQQQQ QQQQQQQQQQ QQQQQQQQQQ QQQQQQQQQQ QQQQQQQQQ	GGGGSGGGGS GGGGSGGGGS	mEos3.1		
S3 A	rhx4585	Q_3N					QQQNQQQNQQ QNQQQNQQQN QQQNQQQNQQ QNQQQNQQQN QQQNQQQNQQ QNQQQNQQQ	GGGGSGGGGS GGGGSGGGGS	mEos3.1		
S3 A	rhx4586	Q_7N					QQQQQQQNQQ QQQQQNQQQQ QQQNQQQQQQ QNQQQQQQQN QQQQQQQNQQ QQQQQNQQQ	GGGGSGGGGS GGGGSGGGGS	mEos3.1		
S4B	rhx3450	$Q_3NQ_3N_2$					QQQNQQQNNQ QQNQQQNNQQ QNQQQNNQQQ NQQQNNQQQN QQQNNQQQNQ QQNNQQQNQ				
S4B	rhx3451	$Q_3NQ_3NQ_3N_2$					QQQNQQQNQQ QNNQQQNQQQ NQQQNNQQQN QQQNQQQNNQ QQNQQQNQQQ NNQQQNQQQ				
S4B	rhx3452	$Q_3NQ_3NQ_3NQ_3N_2$					QQQNQQQNQQ QNQQQNNQQQ NQQQNQQQNQ QQNNQQQNQQ QNQQQNQQQN NQQQNQQQN				
S4C	rhx2608	Q_4N_2					QQQQNNQQQQ NNQQQQNNQQ QQNNQQQQNN QQQQNNQQQQ NNQQQQNNQQ QQNNQQQQN	EAAAREAAAR EAAAREAAAR	mEos3.1		
S4C	rhx3789	Q_6N_2					QQQQQQNNQQ QQQQNNQQQQ QQNNQQQQQQ NNQQQQQQNN QQQQQQNNQQ QQQQNNQQQ	EAAAREAAAR EAAAREAAAR	mEos3.1		
S4C	rhx2609	Q_8N_2					QQQQQQQQNN QQQQQQQQNN QQQQQQQQNN QQQQQQQQNN QQQQQQQQNN QQQQQQQQN	EAAAREAAAR EAAAREAAAR	mEos3.1		
S1 C	rhx3947	Q_1N_2					NNQNNQNNQN NQNNQNNQNN QNNQNNQNNQ NNQNNQNNQN NQNNQNNQNN QNNQNNQNN	EAAAREAAAR EAAAREAAAR	mEos3.1		
S1 C	rhx4709	Q_3N_2					QQQNNQQQNN QQQNNQQQNN QQQNNQQQNN QQQNNQQQNN QQQNNQQQNN QQQNNQQQN	EAAAREAAAR EAAAREAAAR	mEos3.1		
5 A	rhx3458	$(Q_4N)_6(Q_3N)_{15}$					QQQQNQQQQN QQQQNQQQQN QQQQNQQQQN QQQNQQQNQQ QNQQQNQQQN QQQNQQQNQQ QNQQQNQQQN QQQNQQQNQQ QNQQQNQQ	EAAAREAAAR EAAAREAAAR	mEos3.1		
5 A	rhx4612	$(Q_2N)_{10}(Q_3N)_{15}$					QQNQQNQQNQ QNQQNQQNQQ NQQNQQNQQN QQQNQQQNQQ QNQQQNQQQN QQQNQQQNQQ QNQQQNQQQN QQQNQQQNQQ QNQQQNQQ	EAAAREAAAR EAAAREAAAR	mEos3.1		
5 B	rhx4294	Q_3N-oDi					QQQNQQQNQQ QNQQQNQQQN QQQNQQQNQQ QNQQQNQQQN QQQNQQQNQQ QNQQQNQQQN	EAAAREAAAR EAAAREAAAR	mEos3.1	GGGGSGGGGS GGGGSGGGGS	PEDEIAALKK EIAALKQENA ALKQEIAALK KEIAALKQG
5 B	rhx4633	Q_3N-FTH1					QQQNQQQNQQ QNQQQNQQQN QQQNQQQNQQ QNQQQNQQQN QQQNQQQNQQ QNQQQNQQQN	EAAAREAAAR EAAAREAAAR	mEos3.1	GGGGSGGGGS GGGGSGGGGS	MTTASTSQVR QNYHQDSEAA INRQINLELY ASYVYLSMSY YFDRDDVALK NFAKYFLHQS HEEREHAEKL MKLQNQRGGR IFLQDIKKPD CDDWESGLNA MECALHLEKN VNQSLLELHK LATDKNDPHL CDFIETHYLN EQVKAIKELG DHVTNLRKMG APESGLAEYL FDKHTLGDSD NES
5 B	rhx4293	Q_7N-oDi					QQQQQQQNQQ QQQQQNQQQQ QQQNQQQQQQ QNQQQQQQQN QQQQQQQNQQ QQQQQNQQQQ	EAAAREAAAR EAAAREAAAR	mEos3.1	GGGGSGGGGS GGGGSGGGGS	PEDEIAALKK EIAALKQENA ALKQEIAALK KEIAALKQG
5 B	rhx4631	Q_7N-FTH1					QQQQQQQNQQ QQQQQNQQQQ QQQNQQQQQQ QNQQQQQQQN QQQQQQQNQQ QQQQQNQQQQ	EAAAREAAAR EAAAREAAAR	mEos3.1	GGGGSGGGGS GGGGSGGGGS	MTTASTSQVR QNYHQDSEAA INRQINLELY ASYVYLSMSY YFDRDDVALK NFAKYFLHQS HEEREHAEKL MKLQNQRGGR IFLQDIKKPD CDDWESGLNA MECALHLEKN VNQSLLELHK LATDKNDPHL CDFIETHYLN EQVKAIKELG DHVTNLRKMG APESGLAEYL FDKHTLGDSD NES
5B	rhx4242	Q60-oDi					QQQQQQQQQQ QQQQQQQQQQ QQQQQQQQQQ QQQQQQQQQQ QQQQQQQQQQ QQQQQQQQQQ	EAAAREAAAR EAAAREAAAR	mEos3.1	GGGGSGGGGS GGGGSGGGGS	PEDEIAALKK EIAALKQENA ALKQEIAALK KEIAALKQG
5B	rhx4632	Q60-FTH1					QQQQQQQQQQ QQQQQQQQQQ QQQQQQQQQQ QQQQQQQQQQ QQQQQQQQQQ QQQQQQQQQQ	EAAAREAAAR EAAAREAAAR	mEos3.1	GGGGSGGGGS GGGGSGGGGS	MTTASTSQVR QNYHQDSEAA INRQINLELY ASYVYLSMSY YFDRDDVALK NFAKYFLHQS HEEREHAEKL MKLQNQRGGR IFLQDIKKPD CDDWESGLNA MECALHLEKN VNQSLLELHK LATDKNDPHL CDFIETHYLN EQVKAIKELG DHVTNLRKMG APESGLAEYL FDKHTLGDSD NES
S5 C	rhx4584	Q60					QQQQQQQQQQ QQQQQQQQQQ QQQQQQQQQQ QQQQQQQQQQ QQQQQQQQQQ QQQQQQQQQQ	GGGGSGGGGS GGGGSGGGGS	mEos3.1		
S5 C	rhx4345b	oDi-Q60	PEDEIAALKK EIAALKQENA ALKQEIAALK KEIAALKQG	GGGGSGGGGS GGGGSGGGGS	mEos3.1	EAAAREAAAR EAAAREAAAR	QQQQQQQQQQ QQQQQQQQQQ QQQQQQQQQQ QQQQQQQQQQ QQQQQQQQQQ QQQQQQQQQQ				
S5 C,D	rhx4615	Q60-oDi					QQQQQQQQQQ QQQQQQQQQQ QQQQQQQQQQ QQQQQQQQQQ QQQQQQQQQQ QQQQQQQQQQ	GGGGSGGGGS GGGGSGGGGS	mEos3.1	GGGGSGGGGS GGGGSGGGGS	PEDEIAALKK EIAALKQENA ALKQEIAALK KEIAALKQG
S5 D	rhx4619	Q60-oDi{X}					QQQQQQQQQQ QQQQQQQQQQ QQQQQQQQQQ QQQQQQQQQQ QQQQQQQQQQ QQQQQQQQQQ	GGGGSGGGGS GGGGSGGGGS	mEos3.1	GGGGSGGGGS GGGGSGGGGS	PEDEIAALKK EIAALKQENA ALKQEIAAKL EIKAAKLQG
5 C	rhx4071	$(Q_6)4$					QQQQQQGGGQ QQQQQGGGQQ QQQQGGGQQQ QQQ	EAAAREAAAR EAAAREAAAR	mEos3.1		
5 C	rhx4134	$(Q_6)4$ "Q10N"					QQQQQQGGGQ QQNQQGGGQQ QQQQGGGQQQ QQQ	EAAAREAAAR EAAAREAAAR	mEos3.1		

bioRxiv preprint doi: https://doi.org/10.1101/2023.03.20.533418; this version posted July 29, 2023. The copyright holder for this preprint (which was not certified by peer review) is the author/funder, who has granted bioRxiv a license to display the preprint in perpetuity. It is made available under aCC-BY 4.0 International license.

Table S2. Summary of results from publically available APR and aggregation propensity predictor webservers. All methods available as of April 2021.

Application Name	Link	Amino Acid Sequence					Reference
		$Q(60)$	$N(60)$	$Q_3N(60)$	$Q_4N(60)$	$Q_5N(60)$	
AGGRESCAN	http://bioinf.uab.es/aggrescan	non-amyloid	non-amyloid	non-amyloid	non-amyloid	non-amyloid	Chonchillo-Solï¿½ et al. (2007)
WALTZ	https://walktz.switchlab.org	non-amyloid	non-amyloid	non-amyloid	non-amyloid	non-amyloid	Maurer-Stroh et al. (2010)
FoldAmyloid	http://bioinfo.protres.ru/fold-amyloid/	amyloid	amyloid	amyloid	amyloid	amyloid	Garbuzynskiy et al. (2010)
3D Profile (ZipperDB)	http://services.mbi.ucla.edu/zipperdb/	non-amyloid	non-amyloid	non-amyloid	non-amyloid	non-amyloid	Thompson et al. (2006)
Amylogram	http://smorfland.uni.wroc.pl/shiny/AmyloGram	non-amyloid	non-amyloid	non-amyloid	non-amyloid	non-amyloid	Burdukiewicz et al. (2017)
ANuPP	https://web.iitm.ac.in/bioinfo2/ANuPP/	non-amyloid	non-amyloid	non-amyloid	non-amyloid	non-amyloid	Prabakaran et al. (2020)
TANGO	http://tango.crg.es/	non-amyloid	non-amyloid	non-amyloid	non-amyloid	non-amyloid	Fernandez-Escamilla et al. (2004)
SecStr	http://biophysics.biol.uoa.gr/SecStr/	non-amyloid	non-amyloid	non-amyloid	non-amyloid	non-amyloid	Hamodrakas et al. (2007)
BETASCAN	http://betascan.csail.mit.edu/	non-amyloid	non-amyloid	non-amyloid	non-amyloid	non-amyloid	Bryan et al. (2009)
Net-CSSP	http://cssp2.sookmyung.ac.kr/index.html	non-amyloid	non-amyloid	non-amyloid	non-amyloid	non-amyloid	Kim et al. (2009)
AmyloidMutants	http://amyloid.csail.mit.edu/	amyloid	non-amyloid	non-amyloid	non-amyloid	non-amyloid	O'Donnell et al. (2011)
PASTA 2	http://old.protein.bio.unipd.it/pasta2/	non-amyloid	non-amyloid	non-amyloid	non-amyloid	non-amyloid	Walsh et al. (2014)
GAP	http://www.iitm.ac.in/bioinfo/GAP/	amyloid	amyloid	amyloid	amyloid	amyloid	Thangakani et al. (2014)
MetAmyl	http://metamyl.genouest.org/	non-amyloid	non-amyloid	non-amyloid	non-amyloid	non-amyloid	Emily et al. (2013)
Budapest Amyloid Predictor	https://pitgroup.org/bap/	non-amyloid	non-amyloid	non-amyloid	non-amyloid	non-amyloid	Keresztes, et al. (2020)
iAMY-SCM	http://camt.pythonanywhere.com/iAMY-SCM	amyloid	amyloid	amyloid	amyloid	amyloid	Charoenkwan, et al. (2020)
