# Supplementary material for: Pathologic polyglutamine aggregation begins with a self-poisoning polymer crystal
Source: bioRxiv. 2023 Mar 21:2023.03.20.533418. Preprint. [Version 1] doi: 10.1101/2023.03.20.533418 (PMC10055281; doi:10.1101/2023.03.20.533418)
Supplement: Supplementary file 2 [file media-1.pdf]

Figure S1

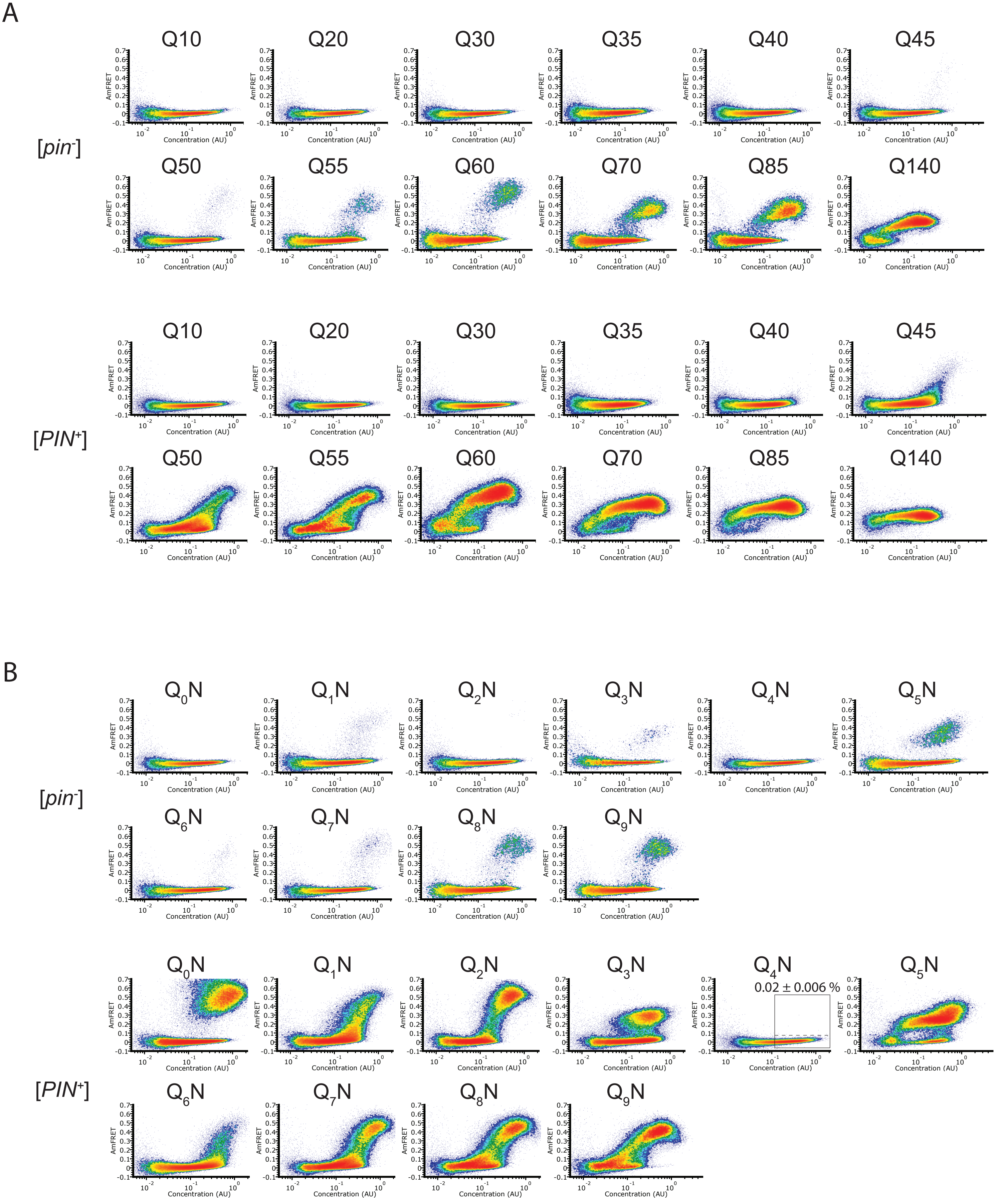
A
[pin-]
Q10
Q20
Q30
Q35
Q40
Q45
Q50
Q55
Q60
Q70
Q85
Q140
[PIN+]
Q10
Q20
Q30
Q35
Q40
Q45
Q50
Q55
Q60
Q70
Q85
Q140
AmFRET
Concentration (AU)
B
[pin-]
Q0N
Q1N
Q2N
Q3N
Q4N
Q5N
Q6N
Q7N
Q8N
Q9N
[PIN+]
Q0N
Q1N
Q2N
Q3N
Q4N
Q5N
Q6N
Q7N
Q8N
Q9N
0.02 ± 0.006 %

Figure S2

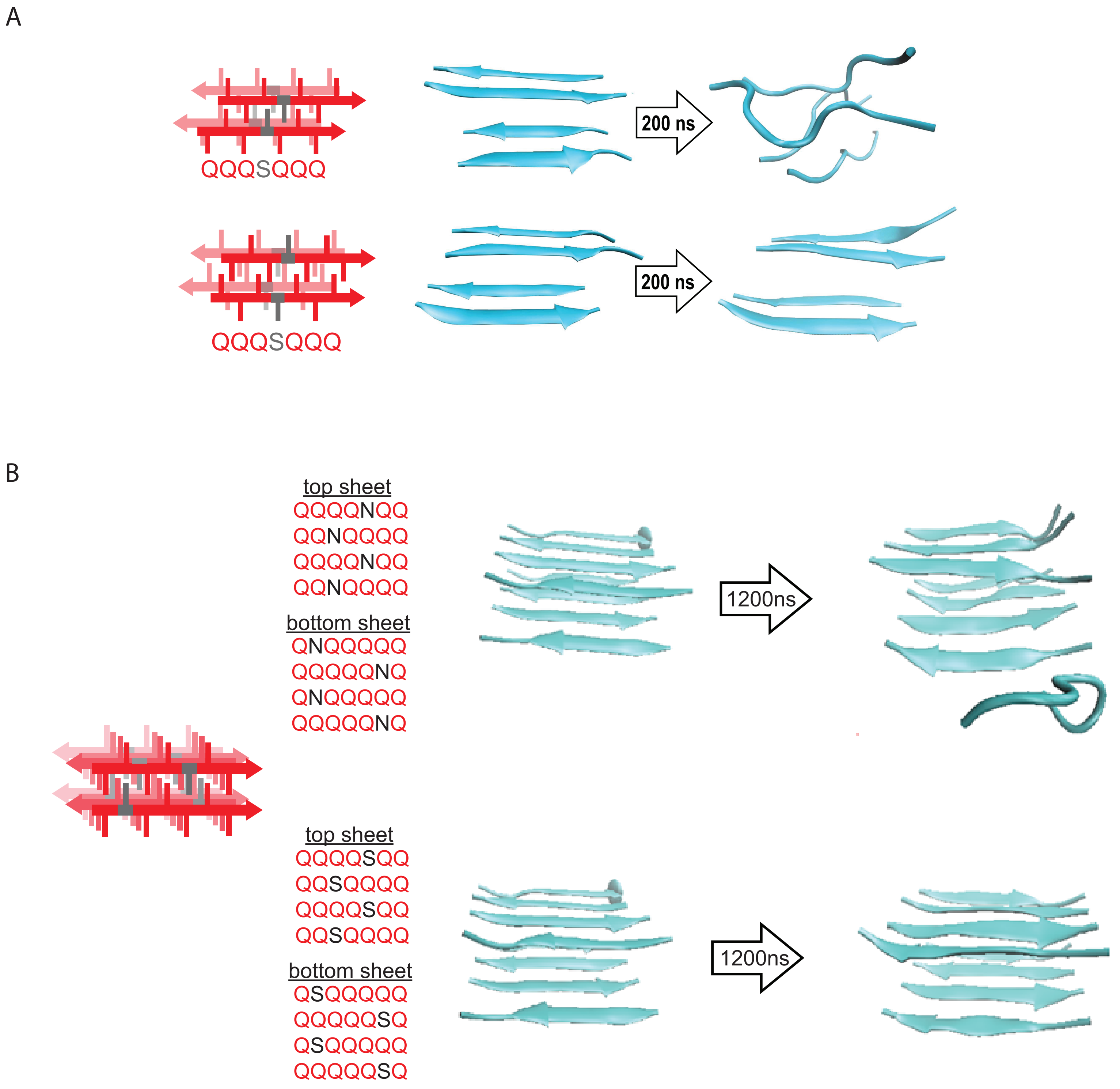
A
QQQSQQQ
200 ns
QQQSQQQ
200 ns
B
top sheet
QQQQNQQ
QQNQQQQ
QQQQNQQ
QQNQQQQ
bottom sheet
QNQQQQQ
QQQQQNQ
QNQQQQQ
QQQQQNQ
1200ns
top sheet
QQQQSQQ
QQSQQQQ
QQQQSQQ
QQSQQQQ
bottom sheet
QSQQQQQ
QQQQQSQ
QSQQQQQ
QQQQQSQ
1200ns

Figure S3

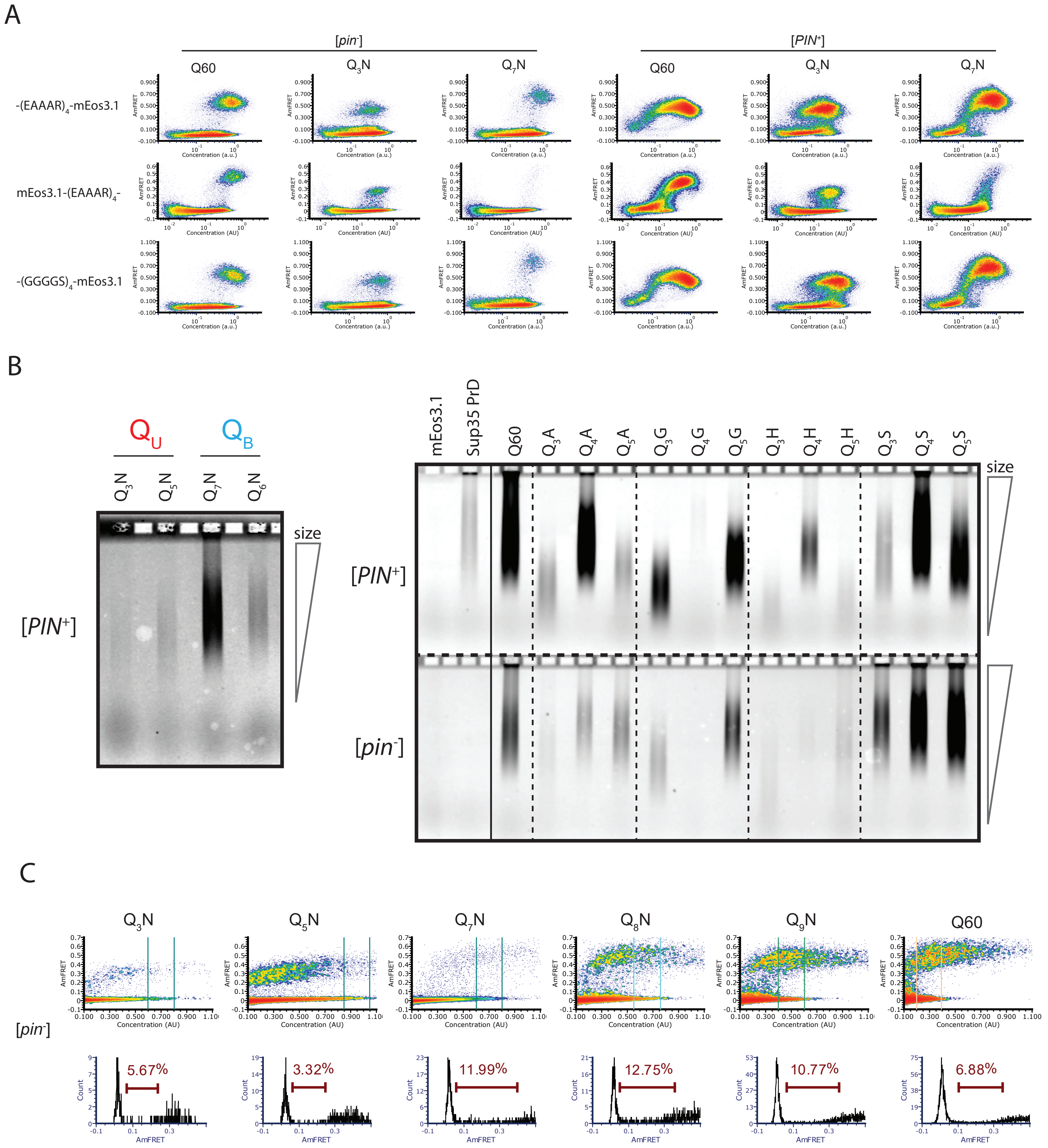
A
[pin-]
[PIN+]
Q60
Q_3N
Q_7N
-$(EAAAR)_4$-mEos3.1
mEos3.1-$(EAAAR)_4$-
-$(GGGGS)_4$-mEos3.1
AmFRET
Concentration (a.u.)
Concentration (AU)
B
Q_U
Q_B
Q_3N
Q_5N
Q_7N
Q_6N
size
[PIN+]
mEos3.1
Sup35 PrD
Q60
Q_3A
Q_4A
Q_5A
Q_3G
Q_4G
Q_5G
Q_3H
Q_4H
Q_5H
Q_3S
Q_4S
Q_5S
size
[PIN+]
[pin-]
C
Q_3N
Q_5N
Q_7N
Q_8N
Q_9N
Q60
[pin-]
AmFRET
Concentration (AU)
Count
AmFRET
5.67%
3.32%
11.99%
12.75%
10.77%
6.88%

Figure S4

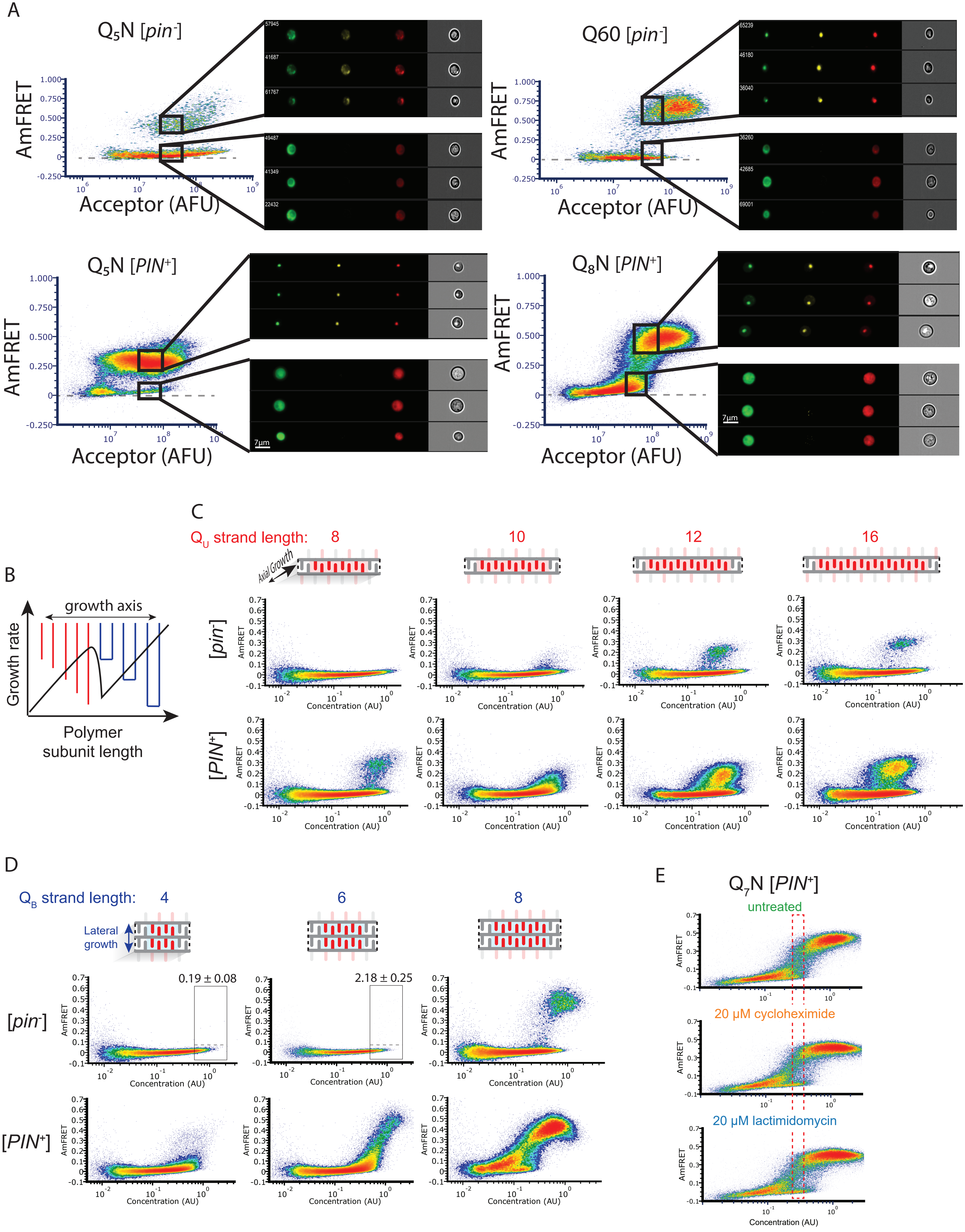
A
Q5N [pin-]
Q60 [pin-]
Q5N [PIN+]
Q8N [PIN+]
AmFRET
Acceptor (AFU)
7µm
B
growth axis
Growth rate
Polymer subunit length
C
QU strand length: 8
10
12
16
Axial Growth
[pin-]
[PIN+]
AmFRET
Concentration (AU)
D
QB strand length: 4
6
8
Lateral growth
0.19 ± 0.08
2.18 ± 0.25
[pin-]
[PIN+]
AmFRET
Concentration (AU)
E
Q7N [PIN+]
untreated
20 µM cycloheximide
20 µM lactimidomycin
AmFRET
Concentration (AU)

Figure S5

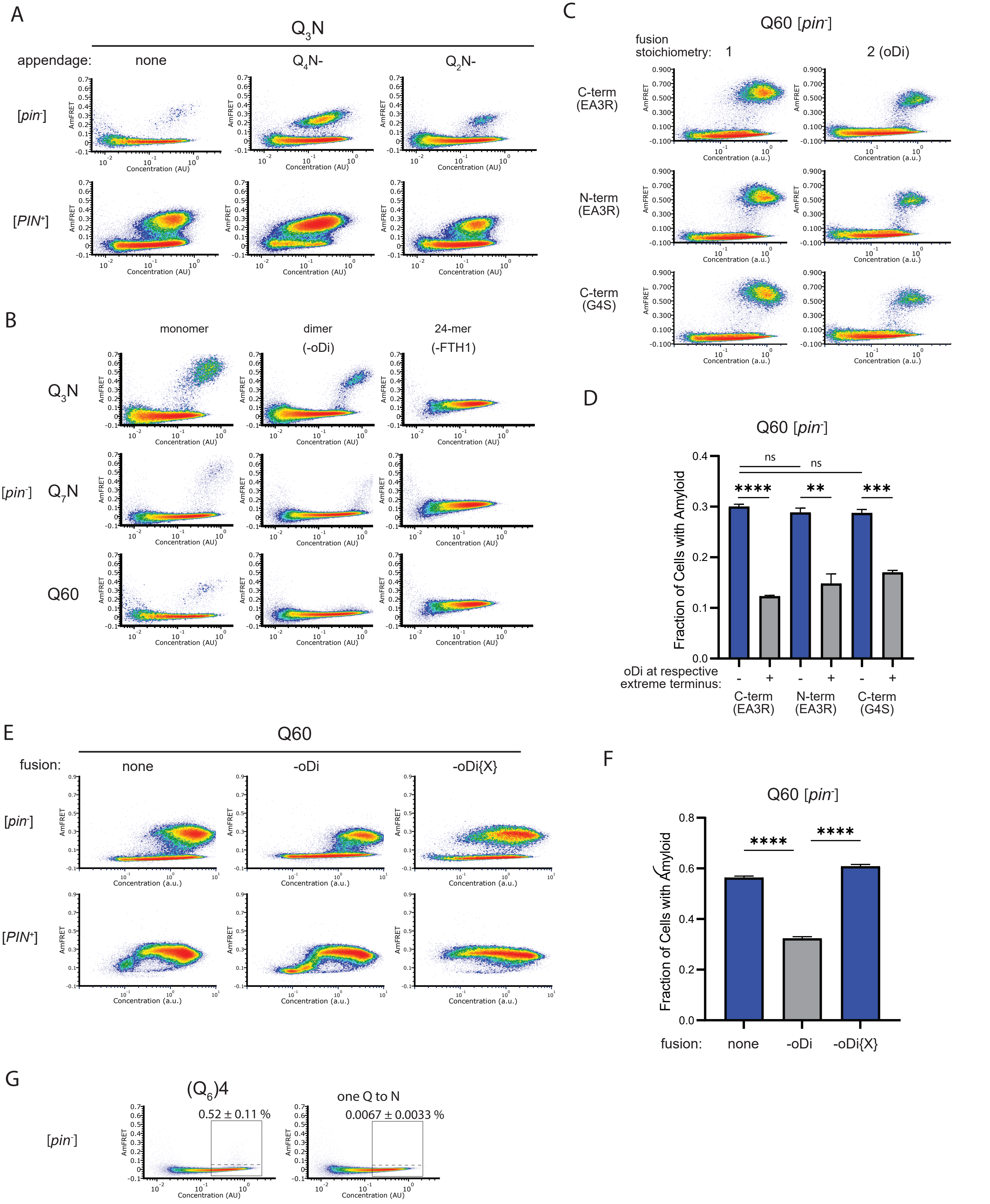
A
Q_3N
appendage:
none
Q_4N-
Q_2N-
[pin-]
[PIN+]
AmFRET
Concentration (AU)
B
monomer
dimer
(-oDi)
24-mer
(-FTH1)
Q_3N
[pin-]
Q_7N
Q60
C
Q60 [pin-]
fusion
stoichiometry:
1
2 (oDi)
C-term
(EA3R)
N-term
(EA3R)
C-term
(G4S)
Concentration (a.u.)
D
Q60 [pin-]
Fraction of Cells with Amyloid
ns
ns
****
**
***
oDi at respective
extreme terminus:
-
+
C-term
(EA3R)
N-term
(EA3R)
C-term
(G4S)
E
Q60
fusion:
none
-oDi
-oDi{X}
[pin-]
[PIN+]
F
Q60 [pin-]
Fraction of Cells with Amyloid
****
****
fusion:
none
-oDi
-oDi{X}
G
$(Q_6)4$
one Q to N
0.52 ± 0.11 %
0.0067 ± 0.0033 %
[pin-]
AmFRET
Concentration (AU)
